# Cyclin-dependent kinase inhibitors exert distinct effects on patient-derived 2D and 3D glioblastoma cell culture models

**DOI:** 10.1038/s41420-021-00423-1

**Published:** 2021-03-15

**Authors:** Christin Riess, Dirk Koczan, Björn Schneider, Charlotte Linke, Katharina del Moral, Carl Friedrich Classen, Claudia Maletzki

**Affiliations:** 1 University Children’s Hospital, Rostock University Medical Centre, Ernst-Heydemann-Straße 8, 18057 Rostock, Germany; 2 Department of Medicine Clinic III - Hematology, Oncology, Palliative Medicine, Rostock University Medical Center, Rostock University Medical Centre, Ernst-Heydemann-Str. 6, 18057 Rostock, Germany; 3 Core Facility for Microarray Analysis, Institute for Immunology, Rostock University Medical Centre, 18057 Rostock, Germany; 4 Institute of Pathology, Strempelstraße 14, 18055 Rostock, Rostock University Medical Centre, 18057 Rostock, Germany

**Keywords:** Targeted therapies, CNS cancer

## Abstract

Current therapeutic approaches have met limited clinical success for glioblastoma multiforme (GBM). Since GBM harbors genomic alterations in cyclin-dependent kinases (CDKs), targeting these structures with specific inhibitors (CDKis) is promising. Here, we describe the antitumoral potential of selective CDKi on low-passage GBM 2D- and 3D models, cultured as neurospheres (NSCs) or glioma stem-like cells (GSCs). By applying selective CDK4/6i abemaciclib and palbociclib, and the more global CDK1/2/5/9-i dinaciclib, different effects were seen. Abemaciclib and dinaciclib significantly affected viability in 2D- and 3D models with clearly visible changes in morphology. Palbociclib had weaker and cell line-specific effects. Motility and invasion were highly affected. Abemaciclib and dinaciclib additionally induced senescence. Also, mitochondrial dysfunction and generation of mitochondrial reactive oxygen species (ROS) were seen. While autophagy was predominantly visible after abemaciclib treatment, dinaciclib evoked γ-H2AX-positive double-strand breaks that were boosted by radiation. Notably, dual administration of dinaciclib and abemaciclib yielded synergistic effects in most cases, but the simultaneous combination with standard chemotherapeutic agent temozolomide (TMZ) was antagonistic. RNA-based microarray analysis showed that gene expression was significantly altered by dinaciclib: genes involved in cell-cycle regulation (different CDKs and their cyclins, *SMC3*), mitosis (*PLK1, TTK*), transcription regulation (*IRX3, MEN1*), cell migration/division (*BCAR1*), and E3 ubiquitination ligases (*RBBP6, FBXO32*) were downregulated, whereas upregulation was seen in genes mediating chemotaxis (*CXCL8, IL6, CCL2*), and DNA-damage or stress (*EGR1, ARC, GADD45A/B*). In a long-term experiment, resistance development was seen in 1/5 cases treated with dinaciclib, but this could be prevented by abemaciclib. Vice versa, adding TMZ abrogated therapeutic effects of dinaciclib and growth was comparable to controls. With this comprehensive analysis, we confirm the therapeutic activity of selective CDKi in GBM. In addition to the careful selection of individual drugs, the timing of each combination partner needs to be considered to prevent resistance.

## Introduction

Cyclin-dependent kinases (CDKs) play indispensable roles in a variety of biological processes, including cell-cycle control, oncogenic transcription, DNA-damage repair, and stem cell self-renewal^1,2^. In most cancers, genomic alterations in specific CDKs either result in constitutive activation or loss of endogenous modulators, including those of the p16/CDK4‐Cyclin-D/pRb pathway^3^. This imbalance pushes cell-cycle progression and malignant transformation^2^. CDK inhibitors (CDKi’s) specifically targeting these proteins are widely applied in (pre-)clinical oncological research^1,4–8^. CDKi has synergistic activity when applied in conjunction with other targeted drugs, such as BRAF and MEK inhibitors for malignant melanomas^3^. Most clinical trials have confirmed manageable toxicity profiles, with clinical responses in many cases and even significantly prolonged overall survival in selected patients cohorts^9^. To date, the three FDA-approved CDK4/6i abemaciclib, palbociclib, and ribociclib are a front-line treatment in combination with hormonal therapy for metastatic HR^+^-HER2^−^ breast cancer (BC)^9,10^. Numerous ongoing clinical phase II and III studies evaluate the therapeutic potential in other entities. Functionally, CDK4/6 are cell-cycle-regulatory proteins that initiate the G_1_–S-phase transition by interaction with D-type cyclins and regulating Rb phosphorylation to activate or repress gene transcription^2,11^.

GBM is the most common and aggressive primary brain tumor^12^. Current therapeutic approaches using surgery and combined radio-/chemotherapy have met limited clinical success, contributing to the extremely poor 5-year survival rate of <3%^13,14^. Genomic analysis revealed alterations in the p16/CDK4‐Cyclin-D/pRb pathway^13^ as well as specific interphase CDKs, namely CDK1 and CDK5. The latter is strongly associated with tumor initiation^15^. As for GBM, a few studies have investigated the potential of CDKi’s. Raub et al. described the antitumor activity of abemaciclib in an orthotopic glioblastoma rat model showing promising effects that were additive in combination with TMZ^16^. Another recent study even recommended nanoparticle encapsulated with dinaciclib in combination with radiation therapy for GBM via targeting tumor-associated macrophages^17^.

Here, we report the successful elimination of GBM cells by CDKi application with several morphology changes, including cell differentiation and vacuolization. We show that abemaciclib and the more global acting CDKi dinaciclib have individual effects on patient-derived 2D- and 3D models that result in senescence, autophagy, and mitochondrial impairment. By performing long-term in vitro treatment, developing resistance against dinaciclib can be prevented by abemaciclib, but not chemotherapy. These results are highly encouraging to move forward with this strategy.

## Results

### CDKi treatment impairs viability in 2D- and 3D-cultured GBM cells

In a preliminary pilot experiment, we applied the three CDKi’s dinaciclib, abemaciclib, and palbociclib in mono- and combination therapy with TMZ/radiation for 144 h using mostly clinically relevant doses. Abemaciclib is the only exception. Here, higher doses were applied.

**Fig. 1:**
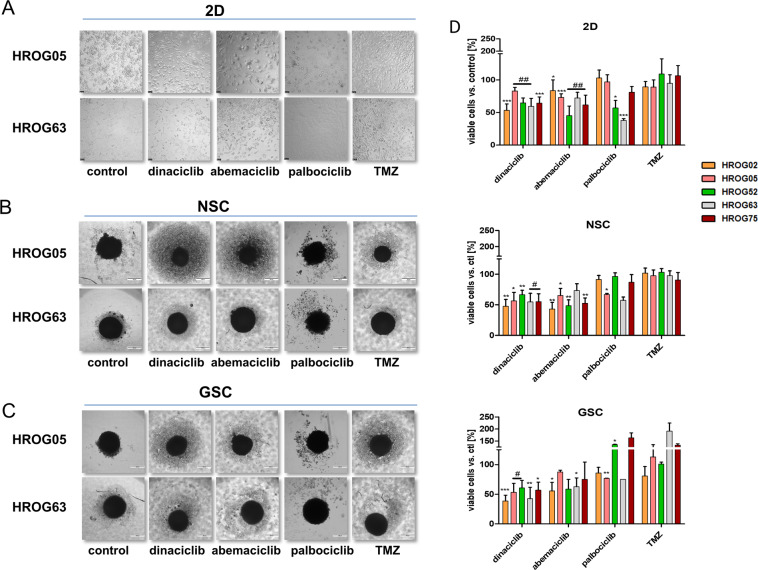
CDKi impairs GBM cell proliferation. GBM cells were treated with the respective substances for 72 h or 2 × 72 h. **A**–**C** Images are representative for the different treatment regimens in HROG05 and HROG63 in 2D- and 3D models (scale bar **A**: 50 μm; **B** and **C**: 200 μm). **D** Quantitative analysis of cell vitality was done via calcein-AM in the 2D model and with 3D-Glo in our 3D systems. Viability reduction (%) after treatment was quantified by normalization to control values (untreated cells set to be 100%). *N* = 5 independent experiments; mean ± SEM, ^∗∗∗^*P* < 0.001; ^∗∗^*P* < 0.01; ^∗^*P* < 0.05 vs. control; ^##^*P* < 0.01; ^#^*P* < 0.05 as indicated by line. Unpaired two-tailed Student’s *t* test was used.

As determined by light microscopy, several morphology changes were observed in GBM 2D cultures after dinaciclib and abemaciclib treatment. Exemplarily shown for HROG05 and HROG63, dinaciclib treatment-induced small vacuoles and cell shrinkage. Abemaciclib-treated cells were enlarged, accompanied by a flattened structure and a striking multivacuolar phenotype (Fig. 1A). Notably, such morphological changes were seen in all cell lines and resulted in reduced cell viability (Fig. 1D). Palbociclib was less effective and TMZ treatment had no impact on viability and morphology at the doses used.

Then, we performed simultaneous and sequential combination regimens (72 h each, dose: IC_20_). The simultaneous treatment describes the concomitant administration of two substances, whereas the sequential regimen is characterized by consecutive administration of the respective drugs. In this comparative setting, only simultaneous, but not sequential treatment with dinaciclib and abemaciclib synergistically potentiated antitumor effects of the monotherapy in 3/5 cases (Supplementary Table 1). With regard to TMZ, a synergistic effect was observed when this drug was added after dinaciclib, while the simultaneous treatment was mostly antagonistic. Combination of abemaciclib and TMZ showed also no benefit (Supplementary Table 1). Dual CDK blockade with dinaciclib and palbociclib was again only antagonistic (Supplementary Table 1).

In the 3D spheroids, cytotoxic effects of CDKi’s were preserved. Still, we observed differences between individual 3D cultures, in which GSCs were more susceptible toward CDKi’s than NSCs (Fig. 1B, C). In detail, dinaciclib and abemaciclib impaired GSC and NSC morphology, contributing to a significantly reduced viability (Fig. 1D). Here again, palbociclib had cell line-specific and only minor impact on viability. TMZ did not have any effect on 3D cultures (Fig. 1B, C). With regard to the combination, we again identified striking differences between simultaneous and sequential regimens and also between individual 3D cultures (Fig. 1D).

To sum up these findings, the timing of each combination partner influences effectiveness. Our results favor the sequential instead of the simultaneous treatment in both 2D- and 3D-cultured GBM cell lines. Also, palbociclib had lower activity against GBM cells than the other CDKi’s dinaciclib and abemaciclib. Consequently, we focused on the latter two agents in further experiments.

### CDKi’s induce apoptotic and necrotic cell death

**Fig. 2:**
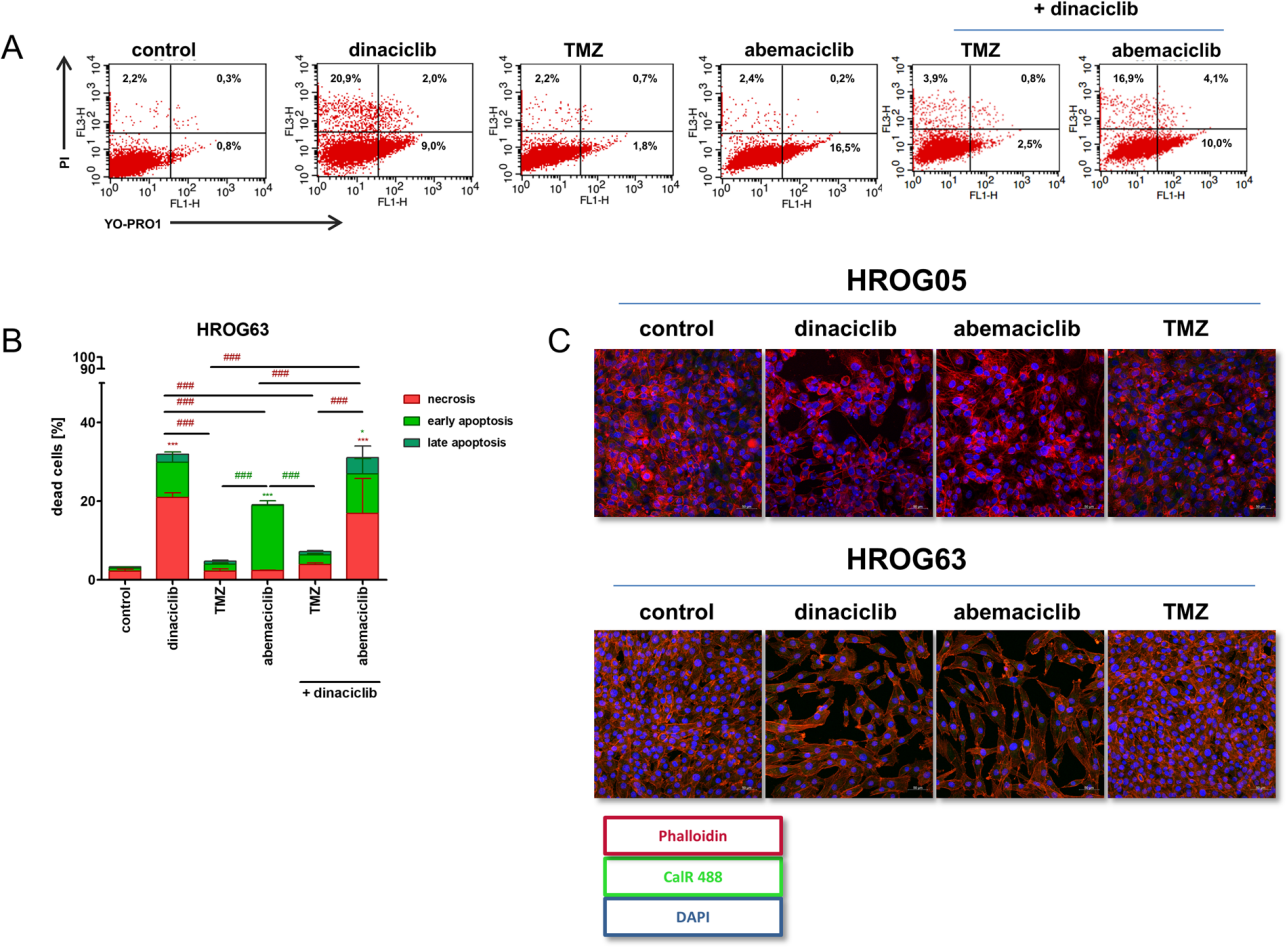
Analysis of cell death induction by CDKi. **A**, **B** Quantitative analysis of cell death using flow cytometric YO-PRO-1/PI staining after incubation with test substances for 72 h in 2D culture. For each sample, 10,000 events were measured. Dead cells were defined as early apoptotic (YO-PRO-1^+^), late apoptotic (YO-PRO-1+/PI^+^), or necrotic (PI^+^). Given are the % numbers of stained cells after treatment. *N* = 3 independent experiments, mean ± SEM, ^∗∗∗^*P* < 0.001; ^∗^*P* < 0.05 vs. control; ^###^*P* < 0.001 as indicated by the line. Two-way ANOVA (Bonferroni’s multiple-comparison test). **C** ICD was detected by using a LSM-780 confocal laser microscope. 2D-cultured cells were stained with anti-Calreticulin Rabbit mAb (Alexa Fluor® 488 Conjugate) and Flash Phalloidin™ Red 594 to visualize surface-exposed CalR and filamentous actin (scale bar **C**: 50 μm).

To describe the effects of CDKi’s in more detail, we then performed flow cytometric apoptosis/necrosis analysis and focused on drug monoapplication. Figure 2 shows HROG63 as an example. Dinaciclib evoked necrosis, abemaciclib triggered early apoptosis (Fig. 2A, B). Dual CDK inhibition induces a mixed response but was not able to enhance cytotoxic effects (Fig. 2A, B). Immunogenic cell death, a common result of CDKi therapy, was not inducible by either treatment (Fig. 2C).

### CDKi’s impair mitochondrial function and evoke methuosis-like processes

**Fig. 3:**
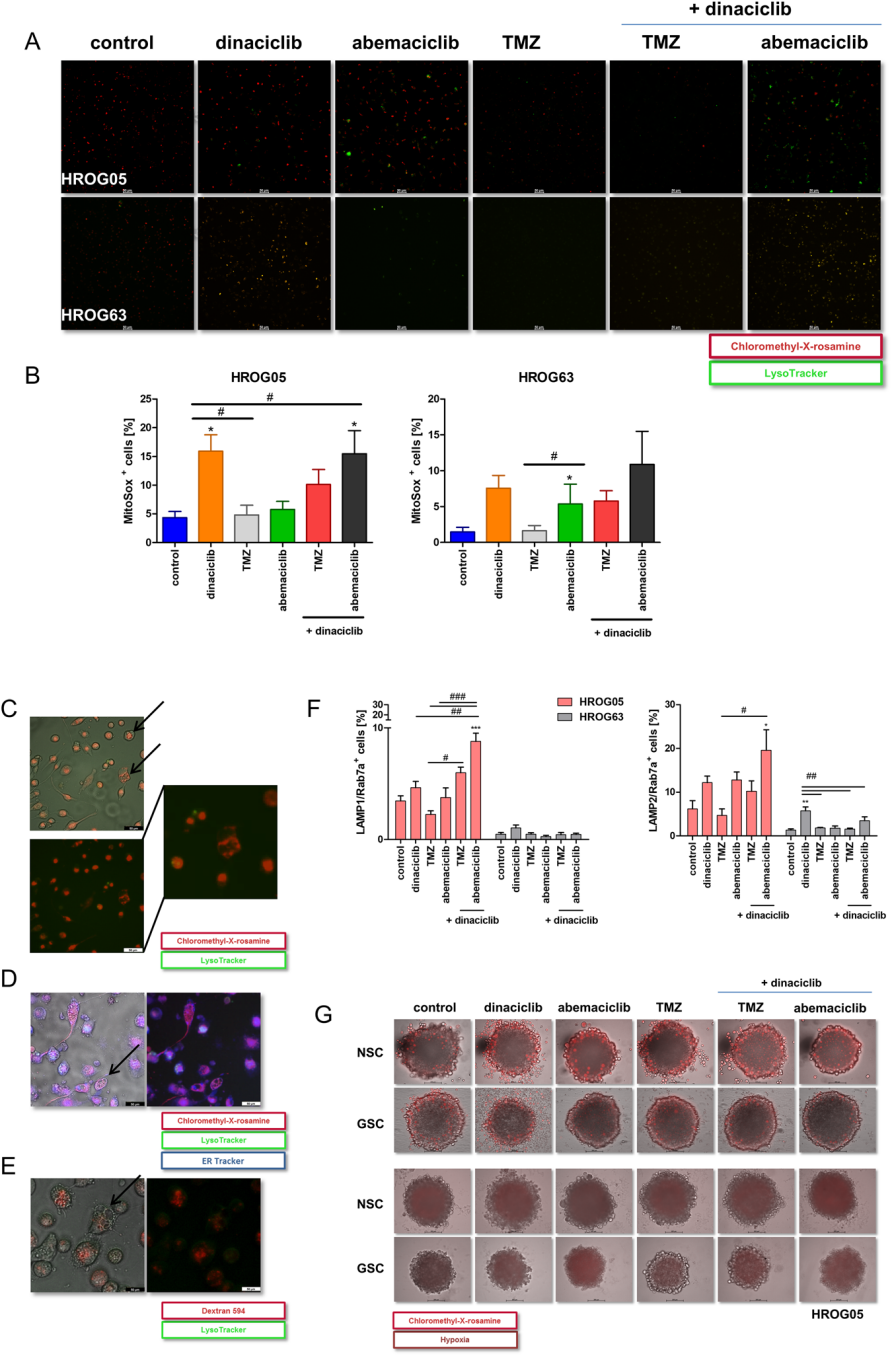
Effects of CDK inhibition on mitochondrial membrane potential and mitochondrial ROS and analysis of vacuole formation by CDKi. **A** 2D-and 3D-cultured GBM cells were treated with CDKi for 72 h and subjected to immunofluorescence imaging as described in “Materials and Methods” (Mito- and Lyso-Tracker). Merged fluorescence is presented (scale bar **A**: 50 μm). **B** Quantitative analysis of mitochondrial ROS. 2D-cultured GBM cells were treated with respective substances for 72 h, stained with MitoSox Red for 30 min, and analyzed by flow cytometry. MitoSox^+^ cells were presented as (%). *N* = 3 independent experiments, mean ± SEM, ^∗^*P* < 0.05 vs. control. ^#^*P* < 0.05 as indicated by line. One-way ANOVA (Bonferroni’s multiple- comparison test) was indicated. **C**–**E** Analysis of vacuole formation. Merged and separated fluorescence/bright-field images are presented (Mito-, Lyso, and ER-Tracker or dextran and LysoTracker). Arrows indicate vacuoles not colocalizing with green (acidic components), blue, and red dots (ER and dextran = endoplasmic origin, MMP = mitochondrial origin) (scale bar **C**–**E**: 50 μm). A high-magnification image is indicated by the two lines. **F** GBM cells were treated as indicated, stained with CD107a/CD107b and Rab7a, and analyzed via flow cytometry. Presented are cells (HROG05, HROG63) positive for LAMP1/Rab7a and LAMP2/Rab7a in percentage. *N* = 3 independent experiments, mean ± SEM, ^∗∗∗^*P* < 0.001; ^∗∗^*P* < 0.01 vs. control. ^###^*P* < 0.001; ^##^*P* < 0.01; ^#^*P* < 0.05 as indicated by line. One-way ANOVA (Bonferroni’s multiple-comparison test) is indicated. **G** 3D-cultured GBM cells as stated in “Materials and Methods”, treated for 72 h and stained with MitoTracker Red (30 min at 37 °C) or Hypoxia IT Image Red (hypoxia reagent was exposed for 3 h) (scale bar **G**: 200 μm).

**Fig. 4:**
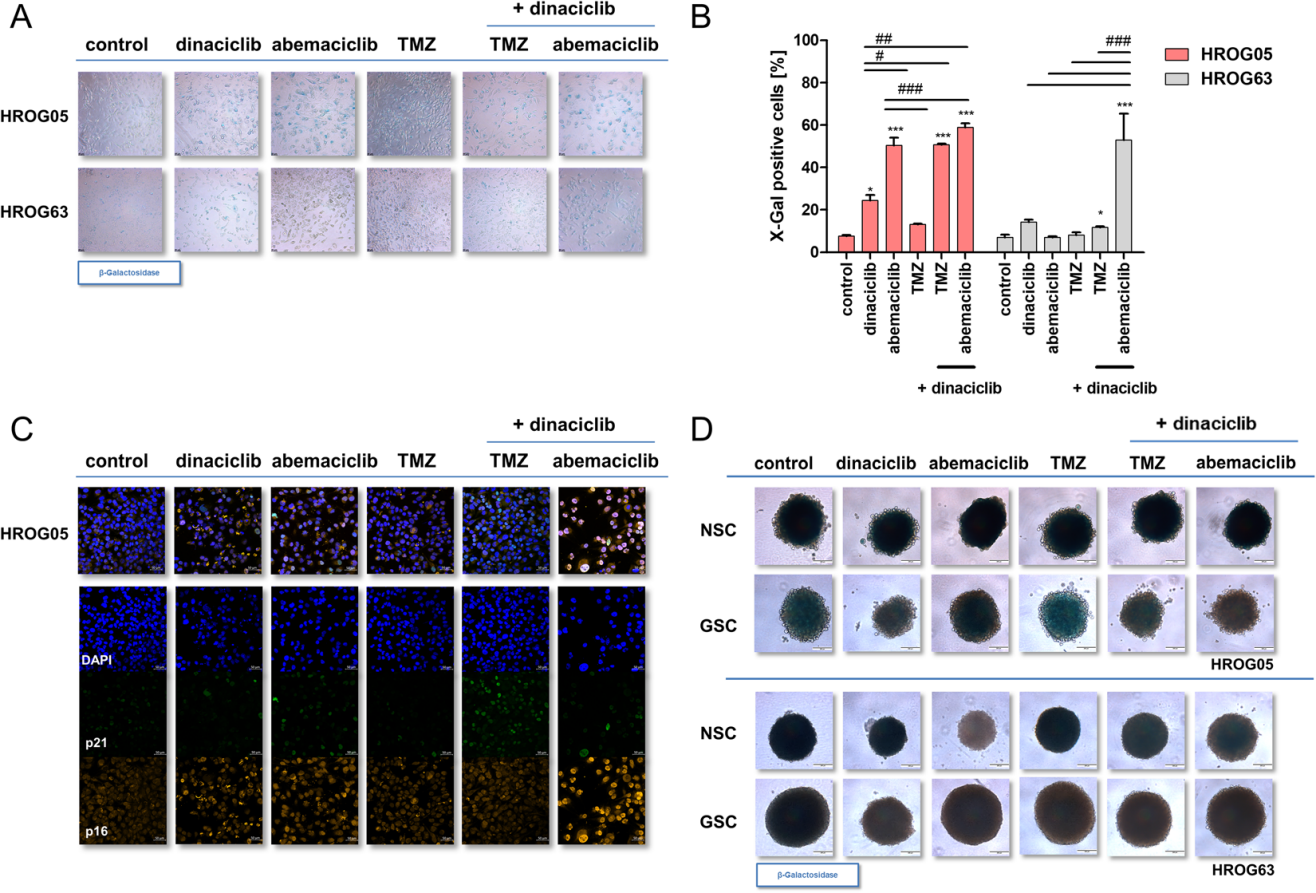
CDK inhibition induces senescence via activation of p16/p21 and ß-galactosidase. 2D- and 3D-cultured GBM cells (**A**, **C**, **D**) were treated with test substances for 72 h, fixed, and stained with β-galactosidase staining solution overnight at 37 °C without CO_2_. Blue: ß-galactosidase activity, indicative for senescence. Representative images are shown (scale bar: **A**: 50 μm; **C**: 200 μm). **B** Quantitative analyses of X-Gal-positive 2D-cultured cells in relation to the whole-cell number/image. **C** Representative images of GBM demonstrate an increase in p16 and p21 after CDK inhibition. The cell was treated as indicated, fixed, permeabilized, and stained with p21 Waf1/Cip1 (12D1) rabbit mAb (Alexa 488 conjugate) (green) and p16 antibody (JC8): sc-56330 Alexa 546 (orange) (scale bar: **D**: 20 μm). *N* = 3 independent experiments, mean ± SEM; ^∗∗∗^*P* < 0.001; ^∗^*P* < 0.05 vs. control; ^###^*P* < 0.001 as indicated by the line. Two-way ANOVA (Bonferroni’s multiple-comparison test).

Then, we analyzed the cause of cytoplasmic vacuole formation after CDKi treatment and screened the GBM cells for autophagy induction. Autophagy is responsible for maintaining cellular homeostasis, but promotes cell death after long-term stress. We indeed observed CDKi-induced lysosomal activation in HROG05 and HROG63, primarily in abemaciclib-treated cells (Fig. 3A). While this was hardly the only explanation for the observed morphology changes, we checked whether CDKi treatment causes mitochondrial damage. The mitochondrial membrane potential (MMP) increased in the 2D culture upon dinaciclib and abemaciclib, indicative of mitochondrial hyperpolarization (Fig. 3A). Such mitochondrial dysfunction may lead to oxidative stress and ROS production. Indeed, mono- and dual-CDKi administration additionally increased mitochondrial reactive oxygen species (mito-ROS) levels (Fig. 3B), which corresponds to an increased MMP. TMZ alone or in combination with dinaciclib did not boost MMP and mito-ROS levels (Fig. 3A, B). Intensified MMP signals were partially accompanied by a higher intensity of the acidic activity. In HROG63 cells, MMP signals and acidic compartments overlapped, while this was not the case in HROG05 (Fig. 4A). Also, CDKi-induced huge vacuoles in HROG05 cells were neither positive for MMP, lysosomal activity, ER-Tracker, nor Dextran (Fig. 3C–E). Hence, CDKi may block the lysosomal processing of these vesicles.

So, we next investigated the origin of vacuole formation. By measuring LAMP-1/-2 and Rab7a via flow cytometry, single- and dual-CDKi application increased the percentage of LAMP1/2-Rab7a-positive cells. The cell line HROG05 showed the most pronounced morphological changes with massive vacuolization and LAMP1/2-Rab7a abundance under CDKi therapy (Fig. 3C–F). These cumulative data are indicative of the induction of early methuosis-like processes.

To see whether similar mechanisms are present in 3D cultures, we next checked mitochondrial activity and hypoxia within NSC and GSC spheres (Fig. 3G). In both culture models, MMP was seen at the edge of the spheres. Mitochondrial hyperpolarization was especially seen after dinaciclib and abemaciclib treatment of NSC. Dual-CDKi treatment and dinaciclib in combination with TMZ accelerated the abundance of MMP signals, which were also visible in the inner sphere (Fig. 3G, upper part). Additional hypoxia analysis revealed that NSC had more hypoxic cores than GSC—irrespective of the applied treatment schedule (Fig. 3G, lower part). Mono- and dual-CDKi application increased hypoxia in these cells. In GSC, hypoxic cores increased after dinaciclib and abemaciclib monotherapy. The combination of CDKi’s or with TMZ did not boost hypoxia significantly. Hence, hypoxia plays a minor role here.

Taken together, CDKi alone or in combination triggers a specific and uncommon mode of cell death that is characterized by a multivacuolar phenotype and signs of early methuosis.

### CDKi’s trigger senescence induction in 2D- and 3D-cultured GBM cell lines

Senescence can be triggered by different damaging stimuli, including telomere shortening (replicative senescence), oxidative DNA damage, and a persistent DNA damage response. Here, senescence is likely provoked by either ROS in hyperpolarized mitochondria (oxidative DNA damage) or accumulation of γH2AX foci that represent a subset of repair-proof lesions that seem to persist (DNA-damage response). Hence, we examined the activity of β-galactosidase, as well as activation of p16/p21 in selected 2D- and 3D models as markers of senescence (Fig. 4). Senescence induction was observed in HROG05 and HROG63 cells, with, however, interindividual differences (Fig. 4A, B). Mono- and dual application of dinaciclib and abemaciclib was most effective. TMZ induced senescence in HROG05 cells, the combination with dinaciclib boosted the effects (Fig. 4A, B). The expression of senescence markers p16 and p21 increased in HROG05 cells after CDKi treatment and underlines the results of the β-galactosidase staining. p16 was detected in the nucleus and cytoplasm of the cells, whereas p21 was only found in the nucleus. Dual application of both CDKi leads to strong nuclear induction of p16. p21 expression was strongly elevated after dinaciclib and TMZ treatment. In some cases, e.g., in the combination regimens, colocalization of both markers was seen (Fig. 4C). Comparable, though less pronounced effects of β-galactosidase staining were observed in 3D cultures and again mainly visible in HROG05 cells (Fig. 4D). To sum up, CDKi’s trigger senescence via activation of p21 and p16.

### CDKi’s interfere with invasiveness and migratory potential of 2D- and 3D-cultured GBM cell lines

In subsequent experiments, the impact of CDKi’s treatment on cell motility as prerequisites for invasiveness and metastasis was studied in 2D- and 3D cultures.

**Fig. 5:**
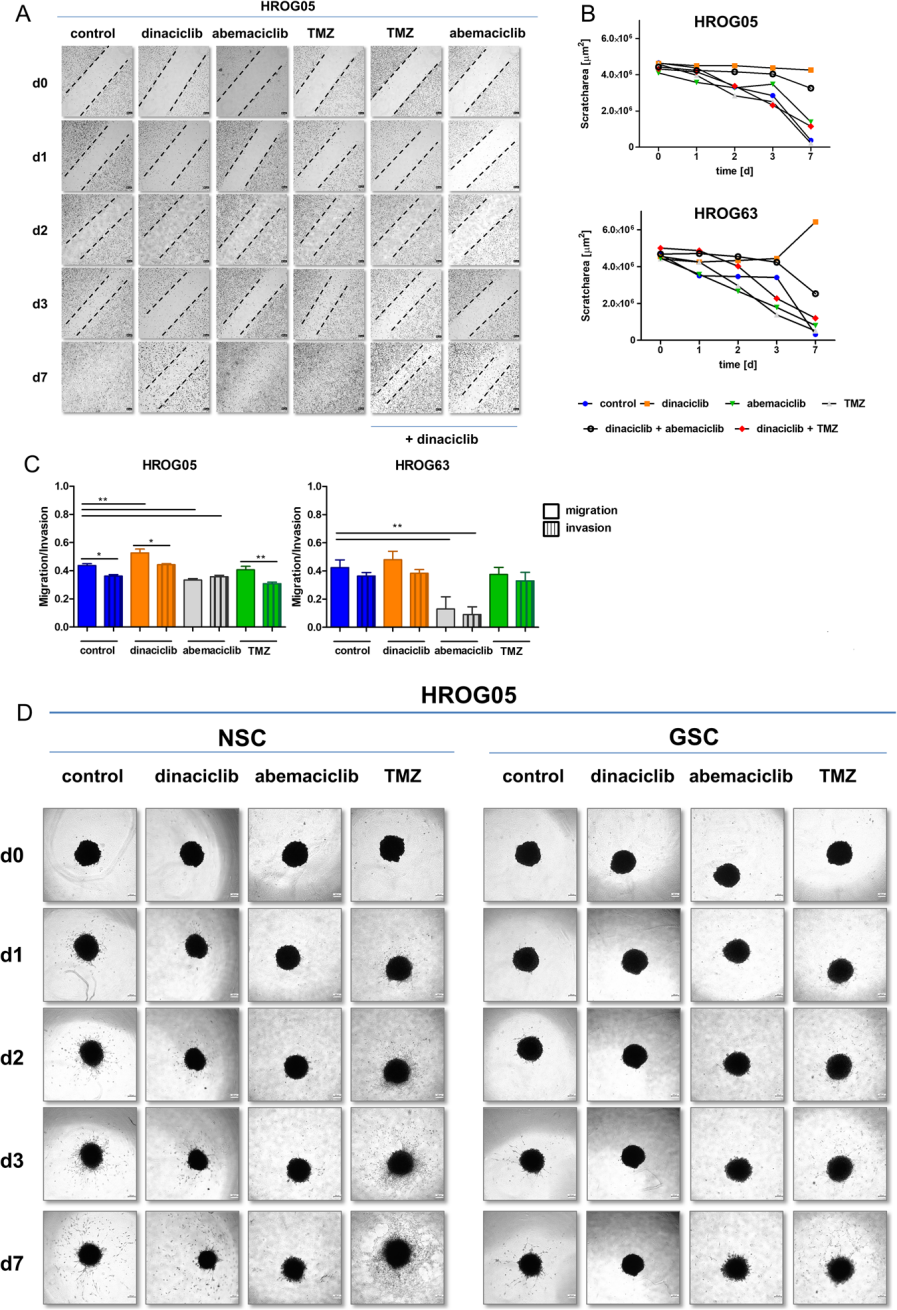
The impact of CDKi on motility, migration, and invasion in 2D- and 3D models. **A**, **B** 2D-cultured GBM cells were seeded and grown to 90% confluency. A standard scratch was placed on the plate utilizing a 200-μl pipette tip. Detached cells were removed, and the above-mentioned substances were added. The scratch closure was monitored for 7 days using a Leica DMI 4000B microscope (scale bar **A**: 50 μm). For manual identification of scratch area (=open wound area), captured images were converted to grayscale. Finally, a threshold, a band-pass, and minimum filter were applied. Region of interest(s) (ROI) could then be set and scratch area analyzed. The scratch area (µm^2^) was plotted over time for each substance. Data are presented as mean. **C** Effects of CDKi on cell invasion and migration assessed by a Boyden chamber assay. *N* = 3 independent experiments, mean ± SEM, ^∗∗^*P* < 0.01; ^∗^*P* < 0.05 as indicated by the line. One-way ANOVA (Bonferroni’s multiple-comparison test). **D** Analysis of CDKi-treated 3D-cultured GBM cell invasion into a Matrigel matrix (scale bar **D**: 250 μm). GBM cells were monitored for a total of 7 days.

First, a wound-healing assay was done. Figure 5A, B shows representative images of 2D-cultured HROG05 cells along with quantified scratch areas. Control and TMZ-treated cells proliferated appeared normal and almost closed the wound within 3 days (scratch completion after 7 days). Dinaciclib completely prevented GBM cell proliferation, which was characterized by cell shrinkage and cell death, leading to scratch areas higher than at day zero (Fig. 5A, B). Abemaciclib treatment decelerated wound healing and scratches did not fully close within the specified time of seven days (Fig. 5A, B). Dual CDKi treatment had comparable effects as the monotherapy; the addition of TMZ had no influence.

In a subsequent Matrigel-based invasion–migration assay, the migration and invasiveness of GBM cells slightly increased under dinaciclib treatment, likely constituting to some kind of escape. Abemaciclib reduced migration/invasion in HROG05 cells and led to a significant 4-fold decrease in HROG63 cells (Fig. 5C, *P* < 0.01 vs. control).

To determine the effect of CDKi on GBM spheroids, we finally implanted defined individual NSC and GSC of cell line HROG05 in Matrigel and monitored sphere outgrowth (Fig. 5D). Control cells showed high basal invasiveness into the Matrigel in which NSCs were much more invasive than GSCs. CDKi treatment with dinaciclib or abemaciclib reduced their invasiveness, in some cases they did not even penetrate into the matrix (Fig. 5D). However, TMZ treatment was not able to prevent invasiveness of spheroids, which was even higher than in the control. The main body of the implanted spheroid (NSC) was partially disintegrated due to the massive penetration into the matrix.

Hence, these findings nicely confirm the therapeutic potential of selective CDKi’s to prevent invasion and migration. However, the addition of TMZ has a minor effect.

### CDKi’s have a minor impact on double-strand breaks and radiosensitivity

**Fig. 6:**
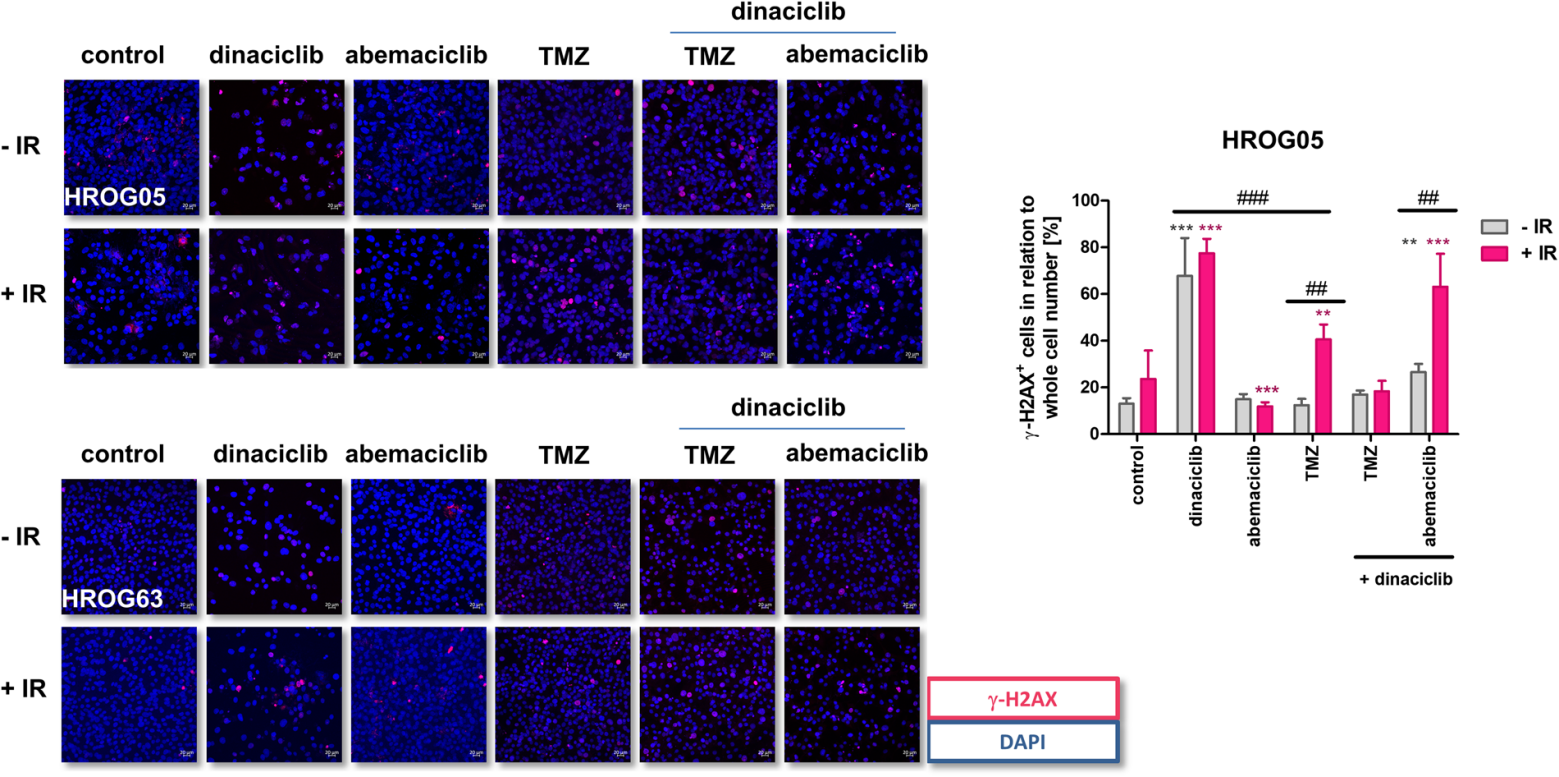
Effect of CDKi on γ-H2AX foci formation in irradiated GBM cells. 2D-cultured cells were seeded on ibidi slides, treated, and irradiated (2 Gy) 24 h after treatment using an IBL 637, followed by 3-day incubation at 37 °C and 5% CO_2_. Cells were incubated with Alexa Fluor® 594 anti-H2A.X Phospho (Ser139) antibody. Nuclei were counterstained with DAPI and analyzed on a confocal laser microscope (scale bar: 20 μm). In addition, nuclei were declaimed as positive if more than 20 foci/nuclei were visible. *N* = 3 independent experiments, mean ± SEM, ^∗∗∗^*P* < 0.001; ^∗∗^*P* < 0.01 vs. control; ^###^*P* < 0.001; ^##^*P* < 0.01 as indicated by line. Two-way ANOVA (Bonferroni’s multiple- comparison test).

Thereafter, we evaluated the effects on signaling and DNA-damage repair. Double-strand breaks (DSB) were determined by γ-H2AX staining, which promotes chromatin remodeling and the assembly of repair proteins (Fig. 6). Monotherapy of 2D-cultured cells with dinaciclib, but not abemaciclib elevated γ-H2AX foci to a degree comparable to TMZ. Dual CDKi treatment or in combination with TMZ was not able to potentiate the effects of the monotherapy.

Then, we examined radiation-induced DSB. Radiation itself had little impact on γ-H2AX foci, but dinaciclib pretreatment boosted DSB. No such radiosensitizing effect was seen for abemaciclib. TMZ likewise induced radiation-induced DSB, but in combination with dinaciclib, this effect completely vanished in HROG05 cells whereas this was not the case in HROG63 cells.

### Microarray analysis identifies molecular alterations upon dinaciclib and confirms the therapeutic activity

**Fig. 7:**
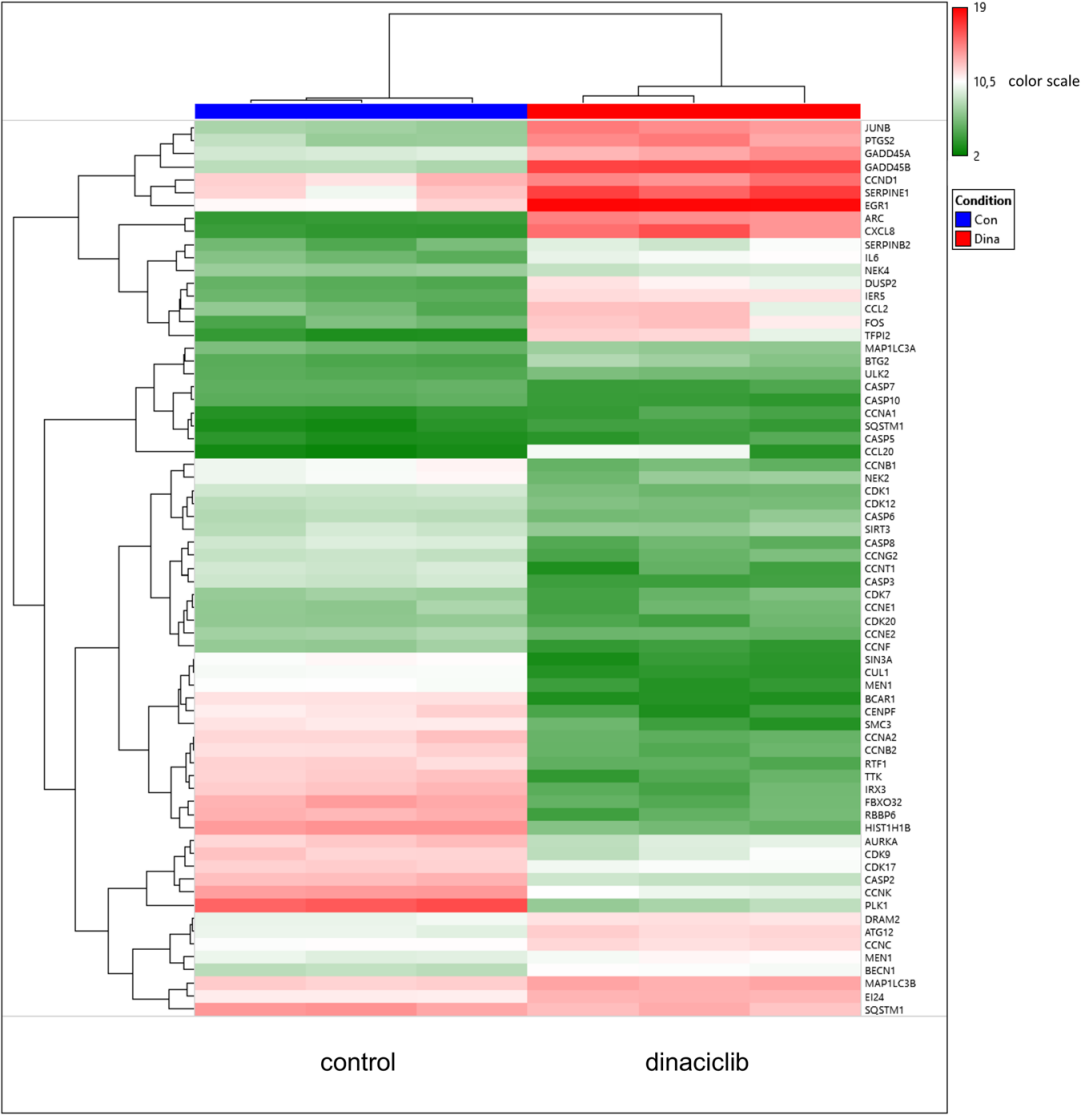
Molecular alterations upon dinaciclib. Heatmap showing RNA expression level from 2D-cultured HROG63 cells assessed by Affymetrix Human Clariom S Array. Primary data analysis was performed with the Affymetrix TAC including the SST-RMA for normalization. Gene expression data were log-transformed. Limma was used here to calculate the p-value. A change was considered significant when the Limma eBayes *P* value met the criterion *P* < 0.05 at fold changes >|2|, i.e., expression increments or declines larger than two. *N* = 3 independent experiments.

As a part of our study, we conducted microarray analyses of 2D-cultured HROG63 cells, either treated with dinaciclib or left untreated (Fig. 7 and Supplementary Table 2). Among the 8008 genes in this analysis having a fold change of 2 and a *P* value of <0.05, 4447 were up- and 3561 downregulated.

The most downregulated genes were *BCAR1*, *PLK1*, *TTK*, *SIN3A*, *CENPF*, and *SMC3*, all being involved in cell-cycle and mitosis inhibition. Genes mediating transcription regulation (*RBBP6, IRX3, RTF1*, and *MEN1*), chromosome remodeling (*HIST1H1B*), and those encoding for E3 ubiquitination ligases (*RBBP6, FBXO32, CUL1*) were also strongly downregulated. The expression levels of *CXCL8, ARC, EGR1, TFPI2, GADD45B, CCL20, EGR1, PTGS2, JUNB, FOS, CCL2, DUSP2, IER5*, and *IL6* mRNA significantly increased. CDKs that were downregulated involved the known targets CDK1 (*CCNA2, B1/2*) and CDK9 *(CCNK, T1)* as well as other CDKs, including CDK7 *(CCNA2, B1/2*, E1), CDK12 *(CCNK)*, CDK17, and CDK20. While senescence was already confirmed by SA-β-Gal staining, senescence-associated genes (S*ERPINB2/E1*, *BTG2*, and *NEK4*) were also elevated. The same applies to genes regulating autophagy and apoptosis. The former were upregulated (*ULK2, MAP1LC3A/B, BECN1, ATG12, DRAM2, SQSTM1*, and *EI24*), the latter (*CASP* genes) showed mostly downregulation. *CASP5* is the only exception.

Taken together, these molecular data nicely underpin our findings on the complex effects of the multi-CDKi dinaciclib on GBM cells.

### Resistance development can be abrogated by combined CDK inhibition

**Fig. 8:**
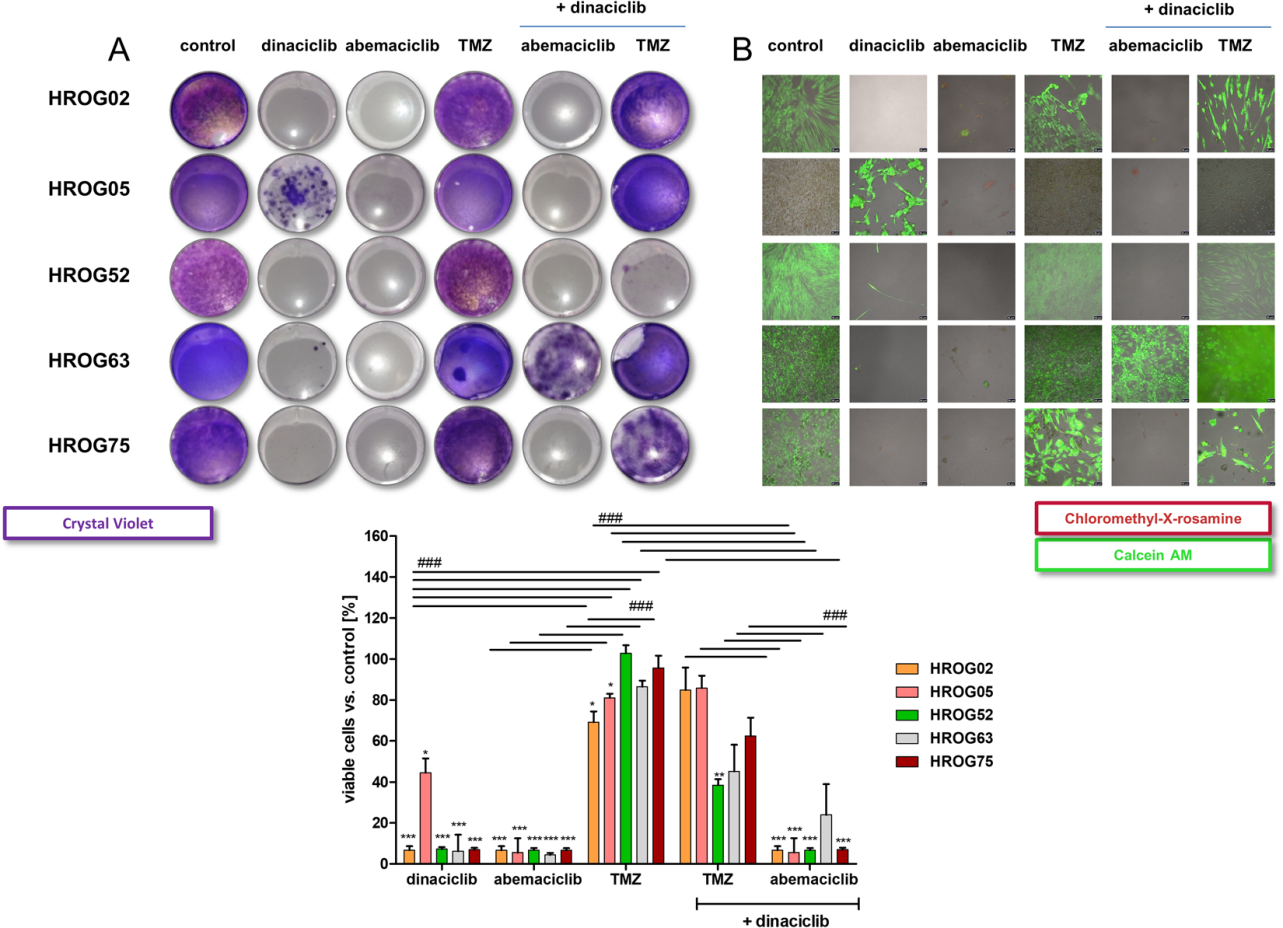
Effects of long-term exposition to CDKi on 2D-cultured GBM cells. **A**, **B** Analysis of cell viability performed by Calcein AM/MitoTracker and crystal violet staining after long-term exposure of respective substances in a ×10 repetitive treatment cycle. Representative images are shown (scale bar **B**: 50 μm). Viability reduction (%) after treatment was quantified by normalization to control values (untreated cells, set to be 100%). *N* = 4 independent experiments, ^∗∗∗^*P* < 0.001; ^∗∗^*P* < 0.01 vs. control; ^###^*P* < 0.001; as indicated by the line. Two-way ANOVA (Bonferroni’s multiple- comparison test).

Finally, we studied resistance development under ongoing treatment. A long-term treatment approach of ten repetitive weekly cycles was carried out on 2D-cultured cells. Crystal-violet and calcein-AM/MitoTracker staining were used to visualize effects (Fig. 8).

Dinaciclib long-term treatment completely inhibited colony formation in 3/5 cases. Interestingly, most colonies were seen in HROG05 cells that already displayed the weakest sensitivity toward dinaciclib in 2D short-term therapy. Hence, is it likely that a pre-existing resistant clone was responsible for outgrowth. The other cell line was HROG63, showing small colonies after dinaciclib long-term exposure. Notably, no colony formation was seen after repetitive abemaciclib treatment (Fig. 8). Hence, we formally confirm the therapeutic activity of this CDKi. As anticipated and in addition to the minor effects after short-term exposure, TMZ did not influence colony formation, with growth comparable to controls. Adding dinaciclib to TMZ prevented colony formation in one case (HROG52), but abrogated the therapeutic effect in the remaining cell lines (Fig. 8). The combination of both CDKi was effective in four cases; still, continued growth was seen in cell line HROG63. It is therefore tempting to speculate that the same clone seen with long-term dinaciclib monotherapy was responsible for colony formation in this setting.

Summing up, these data provide evidence that intrinsic rather than acquired resistance plays a role in CDKi treatment failure.

## Discussion

In this study, we provide functional evidence for the antitumoral effects of mono- and dual treatment with CDKi on low-passage GBM models and identify mechanisms of response. Notably, all patient-derived GBM cell lines tested in this study were sensitive to abemaciclib and dinaciclib, while the overall response to palbociclib was weaker and additionally cell line-specific. Abemaciclib is structurally distinct from palbociclib, with higher selectivity for CDK4 than CDK6 and targeting additional kinases, including GSK3α/β and CAMKII α/β/γ^18^, which may explain the individual effects of the two CDK4/6i seen here. In support of this, we also observed different responses to the CDK4/6i in the 3D-spheroid system. By including two individual 3D-culture models (NSC, GSC) that closely resemble in vivo features, these data favor prospective abemaciclib and dinaciclib application instead of palbociclib in treating GBM patients—also due to the blood–brain-barrier permeability of abemaciclib. To underpin this, the mode of cell death was studied in more detail. Here, we focused on abemaciclib and dinaciclib. While both agents induced autophagy with different intensities, abemaciclib additionally increased early apoptotic cells, while dinaciclib predominantly evoked necrosis. CDKi-mediated apoptosis has been described in various tumor models^19–23^. By contrast, autophagy was only recently described for abemaciclib^24^ and just one publication described the cytoprotective role of autophagy under dinaciclib therapy in NSCLC^25^. Though not analyzed in detail here, LC3B-II aggregation and decreased p62/sequestosome1 expression levels are likely alterations as earlier shown for flavopiridol, another pan-CDKi^26^. In addition to the effects described above, abemaciclib and dinaciclib induced senescence via activation of p16/p21 and likewise evoked DNA-damage response, also confirmed by changes in mRNA expression pattern.

Cell motility and invasion are prerequisites for tumor progression and metastasis. Here, abemaciclib decelerated motility and migration/invasion of GBM cells. Dinaciclib completely prevented GBM motility, but was unable to affect migration/invasion in the 2D system. Other studies already described reduced motility in solid tumors under CDKi treatment. CDK4 inhibition was shown to decrease invasion, metastatic spread, and tumor progression in a RB-high pancreatic ductal adenocarcinoma model^27^. One report even suggested the involvement of CDK5 in the metastatic spread; another trial proposed the contribution of vimentin, Snail, COX-2, and PGE2^27–30^. Strikingly, both CDKi prevented invasiveness of NSC and GSC spheroids. Although the natural invasive behavior was higher in NSC compared to GSC, TMZ equally enhanced invasive growth in both 3D models. In NSC, the main body of the implanted spheroid was partially disintegrated into the matrix. In addition to CDK4 and CDK5, cyclin-B/CDK1 may play a role in tumor cell spreading, motility, and invasion^31,32^. Previous studies have also shown that inhibition of CDK2/9 in triple-negative BC cells and CDK9 inhibition in osteosarcoma cells decreased migration by preventing phosphorylation of CDK-mediated Smad3 and RNA POL-ll in triple-negative BC^33,34^.

We further demonstrated mitochondrial dysfunction characterized by elevated MMP levels and overproduction of mito-ROS. The results from the 2D system were additionally confirmed in 3D models showing elevated MMP upon dinaciclib. While the inner sphere showed less MMP signals, they were visible on the edge of the sphere. Mono- and dual CDKi increased hypoxia in NSC, indicating loss of mitochondrial function mainly in the inner sphere^35,36^.

We, therefore, hypothesize that mono- or dual CDKi treatment increases oxidative stress, induces DSB, and potentiates senescence to trigger cell death^37–42^. While MMP signals and acidic compartments overlapped in 2D-cultured HROG63 cells, this was not the case in cell line HROG05. Intriguingly, we also observed multiple huge vacuoles in CDKi-treated cells. CDKi seemed to trigger the uptake of 10-kDa dextrans and the acidotropic agent in small vacuoles, but rarely in the most enlarged ones. Also, huge vacuoles were insensitive for ER-Tracker and MMP, suggesting vacuoles also did not originate from the mitochondria or endoplasmic reticulum. Here, vacuolization was accompanied by a higher abundance of LAMP1/2 and Rab7a, which are late endosomal markers formed in the early stages of methuosis. Methuosis is a nonapoptotic cell death phenotype defined as the accumulation of large fluid-filled cytoplasmic vacuoles that originate from macropinosomes^43^. Here, these characteristics superficially may resemble methuosis(-like) processes in addition to the appearance of apoptosis^43^. Recent data from the literature support this mechanism, as some antineoplastic agents, including abemaciclib promote vacuolization, which may lead to methuosis^24,44,45^.

GBM cells may have intrinsic and/or acquired radioresistance. Dinaciclib, but not abemaciclib induced γ-H2AX foci that were boosted after combined CDKi radiotherapy. The induction of DSBs was accompanied by downregulation of *CDK1* and *SIRT3*, the latter being known to mediate radioresistance^46^. Hence, dinaciclib reversed radioresistance.

By directly comparing the cytotoxic activity of abemaciclib and dinaciclib, the latter was more potent, likely due to the global activity in targeting multiple CDK. Finally, the strong cytotoxic effects of dinaciclib were confirmed in the microarray data of HROG63 cells. Genes involved in cell-cycle regulation/progression, mitosis, transcription regulation, cell migration/adhesion/division, and those encoding for E3 ubiquitination ligases were strongly downregulated, whereas the expression level of chemotaxis-mediating and DNA-damage or stress genes was significantly upregulated. The upregulation of DDR genes such as *GADD45A/B* which affects aurora-A and Nek2 and therefore promotes genomic instability and histone alterations^47,48^ along with reduced *CDK1* and *SIRT3* expression may explain the treatment-induced accumulation of γ-H2AX. While CDKs are not only involved in cell-cycle progression, they play also crucial roles in neuronal differentiation, transcription regulation, and migration/invasion. Here, several CDKs and their corresponding cyclins are downregulated. Among them, *CDK1* is required for successful completion of M-phase but also contributes to DNA-damage repair, checkpoint activation, and the progression of senescence escape by modulating the survivin pathway in glioma cells^49,50^. Though dinaciclib is widely described as CDK1/2/5/9i, we also detected downregulation of genes encoding for *CDK9*, *CDK12*, and *CDK20* which may have an impact on the protein level. CDK20 promotes cell growth and facilitates radio-chemoresistance in lung cancer cells^51^. CDK12, a transcriptional regulator of homologous recombination that shares sequence homology with CDK9, was previously identified as a target of dinaciclib in BC cells. The therapeutic effect included resensitization to a PARP inhibitor yielding durable regression in a patient-derived xenograft model^52^. Dinaciclib has an even more complex mode of action than previously anticipated. This not only involves cell-cycle arrest, but also cell death via numerous mechanisms: impaired DNA-damage repair, genomic instability, disturbed transcription regulation, and induction of dysregulated mitochondria, senescence, and autophagy. Whether abemaciclib likewise alters gene expression on such a global level, is a matter of speculation and has to be addressed prospectively. Still, our complex set of data indicate that similar mechanisms are altered by abemaciclib. Quite in line, long-term treatment with abemaciclib prevented colony formation even better than dinaciclib. Small viable colonies in some cases might be best explained by single outgrowing clones either intrinsically resistant or rapidly acquiring resistance upon therapeutic pressure. Residual cells showed MMP signals, with reduced viability, indicative of nonintact or almost dying cells.

Summarizing our findings, we show that abemaciclib and dinaciclib, but not palbociclib, inhibited GBM viability/motility, and invasion through multiple mechanisms: senescence, autophagy, necrosis/apoptosis, and mitochondrial dysfunction. We additionally provide mechanistic insights regarding the single-agent activity of dinaciclib. Our data support the idea of using CDKs as therapeutic targets in GBM and suggest dual CDKi application as a new therapeutic approach in clinical trials.

## Materials and methods

### Patient-derived tumor cell lines and culture conditions

Patient-derived GBM cell lines (*N* = 5; HROG02, HROG05, HROG52, HROG63, HROG75) were established in our lab and basically characterized, including molecular analysis and MGMT promoter methylation status^53^. 2D cell cultures were cultured in full medium and incubated at 37 °C in a humidified atmosphere of 5% CO_2_: Dulbecco’s modified eagle medium: nutrient mixture F-12 supplemented with 10% FCS, L-glutamine (2mmol/l), and antibiotics (100 U/ml penicillin/100 μg/ml streptomycin) (all from Pan Biotech, Aidenbach, Germany). 3D GBM neurosphere cells (NSC) and glioma stem-like cells (GSC) were cultured in ultra-low-attachment (ULA) plates (Greiner Bio-One, Kremsmünster, Austria) in a defined medium. NSC was grown in serum-containing medium (=sphere medium), whereas GSC was cultivated in serum-free medium containing stem cell-inducing additives (=stem cell medium) (+ 1× B-27^®^ supplement (50×) (Gibco™, Life Technologies™, Carlsbad, USA), + 20 ng/ml recombinant human epidermal growth factor (rhEGF), + 10 ng/ml bFGF (Immunotools, Friesoythe, Germany). Prior treatment, cells were incubated until spheroids form (~72–96 h). Using this methodology, a single well-defined spheroid with cell line-specific appearance of a particular size was generated.

### Cytostatic drugs and targeted substance

The cytostatics included the CDKi’s (all from Selleckchem, Munich, Germany) abemaciclib (10 µM), palbociclib (10 µM), and dinaciclib (10 or 100 nM). TMZ (10 µM) was obtained from MSD (Haar, Germany). All substances were used in doses below the IC_50_ as determined in the preliminary dose-finding study (range 10 nM –10 µM).

### Viability and senescence assays

Cell viability was assessed by calcein-acetoxymethyl (AM) (Biomol GmbH, Hamburg, Germany) fluorometric assay in 2D culture as described in ref. ^54^. 3D cultures were analyzed luminometric using the CellTiter-Glo^®^ 3D cell viability assay (Promega, Walldorf, Germany) according to the manufacturer’s instruction. CellTiter-Glo^®^ 3D luminescence signal was read with a microplate reader (Infinite^®^ M200, Tecan Group, Switzerland). In addition, ten cycles of single and combined therapy were done in long-term treatment. Readout was done by Calcein AM + MitoTracker Red (Cell Signaling Technology, Frankfurt/Main, Germany) and crystal violet staining (0.2%, Sigma-Aldrich, St. Louis, USA). Senescence-associated β-galactosidase (SA-β-gal, Cell Signaling Technology, Cambridge, UK), as well as apoptosis and necrosis using flow cytometry-based Yo-Pro1/propidium iodide staining, was measured as described in refs. ^54,55^. For p16 and p21 detection, cells were fixed with 2% paraformaldehyde (PFA) w/o methanol (15 min, Thermo Fisher Scientific, Darmstadt, Germany), washed twice, permeabilized, and blocked with 2% BSA, 0.5% Triton X-100 in PBS for 60 min and stained with p21 Waf1/Cip1 (12D1) rabbit mAb (Alexa 488 Conjugate) (green) (1:300, Cell Signaling Technology) and p16 antibody (JC8): sc-56330 Alexa 546 (orange) (1:50, Santa Cruz Biotechnology, Dallas, TX) overnight at 4 °C. Nuclei were counterstained with DAPI and cells were analyzed on a Zeiss LSM-780 Confocal Laser Microscope (Zeiss, Jena, Germany).

### Immunogenic cell death assays

GBM cells were treated for 72 h. Cells were fixed in 4% PFA w/o methanol (30 min)). Surface-exposed Calreticulin (CalR) was detected using an anti-Calreticulin Rabbit mAb (Alexa Fluor^®^ 488 conjugate) (1:50; overnight, Cell Signaling Technology), followed by filamentous actin staining (Flash Phalloidin™ Red 594 (1:20, 20 min, Biolegend). Nuclei were counterstained with DAPI and cells analyzed on a Zeiss LSM-780 confocal laser microscope.

### Cs-137 γ-irradiation

Cs-137 γ-irradiation (2Gy) was performed 24 h after treatment using an IBL 637 (CIS Bio-International, Codolet, France), followed by 3-day incubation at 37 °C and 5% CO_2_. Double-strand breaks (DSB) were assessed with γ-H2AX staining. For the latter, cells were fixed with 4% PFA/PBS, washed twice, permeabilized (0.5% Triton X-100, 15 min), blocked in 1% BSA (45 min), and incubated with Alexa Fluor^®^ 594 anti-H2A.X Phospho (Ser139) antibody (1:1000, overnight, 4 °C) (Biolegend). Nuclei were counterstained with DAPI and analyzed on a Zeiss LSM-780 confocal laser microscope.

### In vitro wound-healing assay and 2D cellular migration/invasion assay

A wound-healing assay was done as described in ref. ^56^, data acquisition was made using a Leica DMI 4000B microscope (Leica, Heidelberg, Germany). Then, a modified Boyden chamber technique (ThinCerts, Greiner Bio-One) with and without Matrigel-coated membranes (Corning, Corning, USA) was applied according to Ramer et al.^57^.

### Tumor spheroid invasion assay

After sphere formation, 96-ULA well plates were placed on ice; half of the medium was removed, and reagents were added at a twofold final concentration (+ EGF = to stimulate invasion) into U-bottom wells containing ice-cold matrigel (Corning). Spheres were monitored for 7 days and images were taken at 24 h to 3-day intervals with a final record on day 7 (Leica DMI 4000B).

### MitoTracker^®^ Red CMXRos, MitoSOX™ Red, LysoTracker™ Green DND-26 ER Tracker ™ Blue-White DPX, and Dextran Alexa Fluor™ 594; 10,000 MW

MitoTracker CMXRos (8-(4’-chloromethyl) phenyl-2,3,5,6,11,12,14,15-octahydro-1 H,4 H,10 H,13 H-diquinolizino-8 H-xanthylium chloride), LysoTracker Green, ER-Tracker™ Blue-White (Dapoxyl) DPX and Molecular Probes™ Dextran, Alexa Fluor™ 594; 10,000 MW, anionic, fixable dyes were prepared according to the manufacturer’s instructions (Cell Signaling Technology, Thermo Fisher Scientific). Mitochondria (MitoTracker Red CMXRos, 20 nM), ER (ER Tracker, 500 nM), and acidic components (LysoTracker, 50 nM) were stained (30 min, 37 °C) 72 h post treatment; Dextran (100 µg/ml) was given simultaneously with treatment and incubated 72 h. Images were taken on fluorescence microscopy (Leica DMI 4000B). MitoSOX™ Red (5 μM, 30 min, 37 °C, Thermo Fisher Scientific) was used as indicator of mitochondrial superoxide and analyzed via flow cytometry (FACS Verse, BD Bioscience, Franklin Lakes, USA).

### LAMP1/2, Rab7a measurement

Vacuole formation was quantified on cells stained with CD107a, CD107b (lysosomal-associated membrane proteins 1 and 2, LAMP1, LAMP2), and GTPase Ras-related protein (Rab7a; 30 min, 4 °C, + intracellular staining) (all purchased from Biolegend). The analysis was done on a FACS Verse.

### Image-iT hypoxia reagent

3D cultures were maintained, plated, and treated as described. Hypoxia reagent (Thermo Fisher Scientific) was added (10 μM, 3 h, 37 °C). The medium was replaced and cells kept in the incubator (24 h). Cells were imaged using a fluorescence microscope (Leica DMI 4000B).

### Microarray analysis of RNA expression profiles

RNA of treated and control HROG63 cells (5 × 10^5^ cells/treatment) were extracted employing RNeasy Plus Kit (Qiagen, Hilden, Germany) according to the manufacturer’s protocol. The total RNA was quantified on a spectrophotometer (NanoDrop 1000, Thermo Fisher Scientific) and integrity confirmed using the Agilent Bioanalyzer 2100 with an RNA Nano chip kit (both from Agilent Technologies, Waldbronn, Germany). Expression profiling was performed by taking advantage of the Affymetrix Human Clariom S Array (Affymetrix/Thermo Fisher Scientific, Santa Clara, USA), which interrogates over 20,000 well-annotated genes. Therefore, the so-called Whole Transcriptome protocol was employed described in ref. ^58^. Primary data analysis was performed with the Affymetrix Transcriptome Analysis Console (TAC) software, including the SST-RMA for normalization. Gene expression data were log-transformed. Limma was used here to calculate the *P* value. A change was considered significant when the Limma eBayes *P* value met the criterion *P* < 0.05 at fold changes >|2|, i.e., expression increments or declines larger than two.

### Image processing

Quantification of the images was done by using the FIJI-ImageJ software as follows:

Staining intensity was determined by dividing the channels into red, green, and blue. Subsequently, integrated density profiles of the same size were measured in the respective channels.

For manual identification of scratch area (= open wound area), captured images were converted to grayscale. Finally, a threshold, a band-pass, and minimum filter were applied. Region of interest(s) (ROI) could then be set and scratch area analyzed. The scratch area [µm^2^] was plotted over the time for each substance. Data are presented as mean.

### Statistics

All values are given as mean ± SEM. After proving the assumption of normality, differences between controls and treated cells were determined by using the unpaired Student’s *t*-test viability determination. In the case of multiple comparisons, one- or two-way *ANOVA* on ranks (Bonferroni’s multiple-comparison test) was applied. Statistical evaluation was performed using GraphPad PRISM software, version 5.02 (GraphPad Software, San Diego, CA, USA). The criterion for significance was taken to be *P* < 0.05.

## Supplementary information

STable 1

STable 2

STable 3

STable legends
