# Supplementary figures and images for: Cyclin-dependent kinase inhibitors exert distinct effects on patient-derived 2D and 3D glioblastoma cell culture models
Source: Cell Death Discov. 2021 Mar 15;7:54. doi: 10.1038/s41420-021-00423-1 (PMC7961149; doi:10.1038/s41420-021-00423-1)

**Stable 1: Bliss independence calculation.**

**
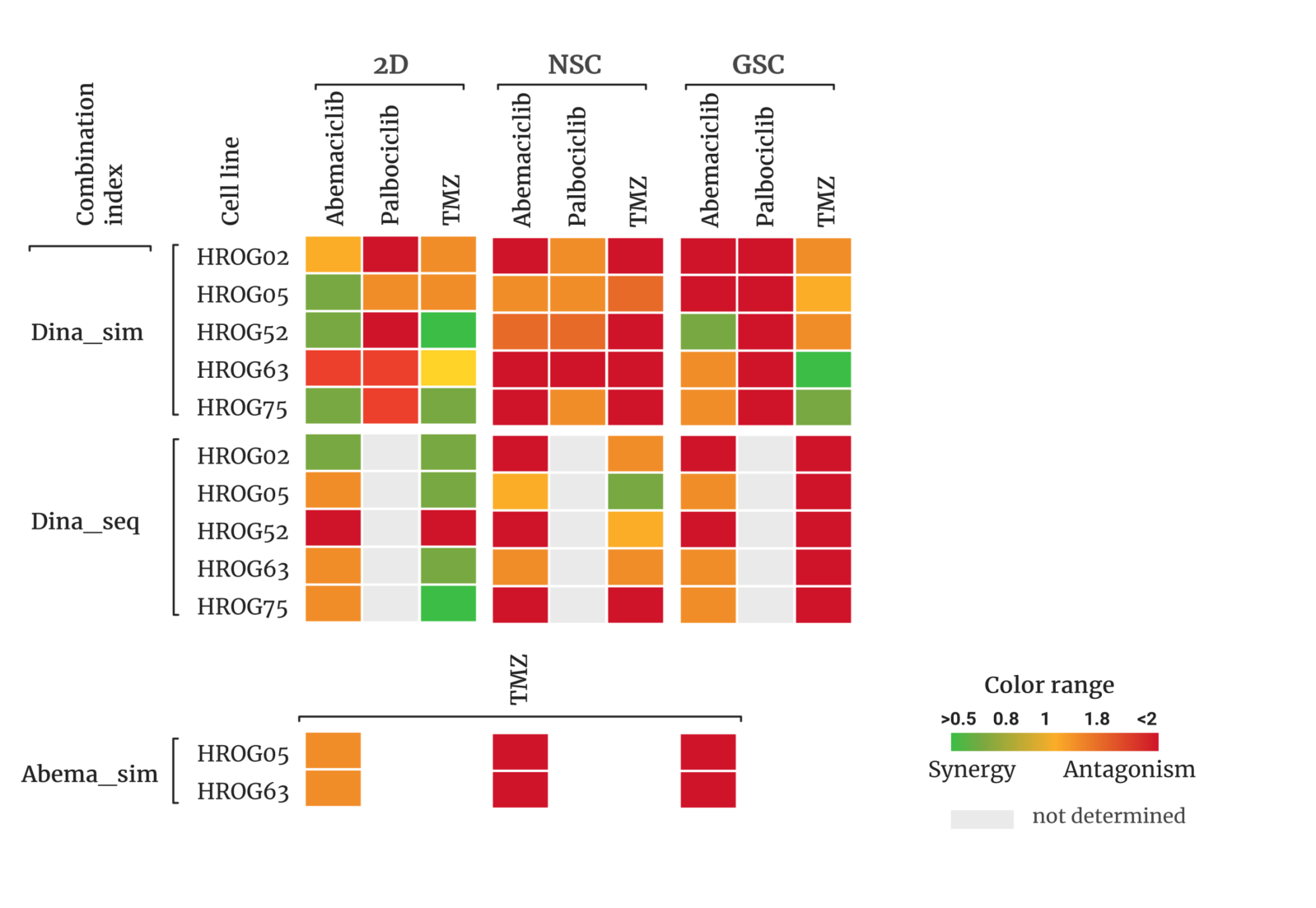
**

Supplement: Supplementary file 1 — STable 1 [file 41420_2021_423_MOESM1_ESM.docx]
