# Supplementary material for: Cyclin-dependent kinase inhibitors exert distinct effects on patient-derived 2D and 3D glioblastoma cell culture models
Source: Cell Death Discov. 2021 Mar 15;7:54. doi: 10.1038/s41420-021-00423-1 (PMC7961149; doi:10.1038/s41420-021-00423-1)
Supplement: Supplementary file 3 — STable 3 [file 41420_2021_423_MOESM3_ESM.docx]

**Cyclin-dependent kinase inhibitors exert distinct effects on patient-derived 2D and 3D glioblastoma cell culture models**

Christin Riess, Dirk Koczan, Björn Schneider, Charlotte Linke, Katharina del Moral, Carl Friedrich Classen, Claudia Maletzki

**Supplementary Information**

Supplementary Table Legends

**Supplementary Table 1 (STable 1).** Bliss independence calculation. The Bliss independence model was calculated based on the 2x72h viability assay on simultaneous and sequential treatment (N=5). If Δ>1, substances were synergistic (green pattern); Δ=1, substances were additive (yellow pattern); Δ<1, substances were antagonistic (orange and red pattern). Grey pattern were determined as “not determined”. Image was created with Biorender.com.

**Supplementary Table 2 (STable 2).** List of differentially expressed genes in HROG63 dinaciclib treated cells compared to HROG63 control cells (Public Gene IDs; ID; Entrez ID; Dina Avg (log2); Con Avg (log2); Fold Change (FC); P-val; gene symbol; chromosome and description). RNA expression level assessed by Affymetrix Human Clariom S Array. Primary data analysis was performed with the Affymetrix TAC including the SST-RMA for normalization. Gene expression data were log-transformed. Limma was used here to calculate the p-value. A change was considered significant when the Limma eBayes P-value met the criterion P < 0.05 at fold changes >|2|, i.e., expression increments or declines larger than two. N = 3 independent experiments.

**Supplementary Table 3 (STable 3).** List of antibodies used in this study (molecule, Cat number, company, species and dilution).
