# Supplementary material for: Cyclin-dependent kinase inhibitors exert distinct effects on patient-derived 2D and 3D glioblastoma cell culture models
Source: Cell Death Discov. 2021 Mar 15;7:54. doi: 10.1038/s41420-021-00423-1 (PMC7961149; doi:10.1038/s41420-021-00423-1)
Supplement: Supplementary file 4 — STable legends [file 41420_2021_423_MOESM4_ESM.docx]

Table S3. Antibodies used in this study.

| Molecule | Cat number | Company | Species | Dilution |
| --- | --- | --- | --- | --- |
| p16 | sc-56330 AF546 | Santa Cruz | Ms | x 50 |
| p21 | 5487S | CST | Rb | x 300 |
| Calreticulin | 62304S | CST | Rb | x 50 |
| γ-H2A.X (Ser139) | 613410 | biolegend | Ms | x 1000 |
| CD107a | 328610 | biolegend | Ms | x 40 |
| CD107b | 354312 | biolegend | Ms | x 40 |
| Rab7a | 850406 | biolegend | Rt | x 50 |
| CST, Cell Signaling Technology; Rb, rabbit; Ms, mouse; Rt, rat | | | | |
